# PamulDB: a comprehensive genomic resource for the study of human- and animal-pathogenic *Pasteurella multocida*

**DOI:** 10.1093/database/baz025

**Published:** 2019-02-25

**Authors:** Tian Li, Xiao-Fei Xu, Hui-Hui Du, Li Li, Neng-Zhang Li, Ze-Yang Zhou, Yuan-Yi Peng

**Affiliations:** 1 State Key Laboratory of Silkworm Genome Biology, Southwest University, Chongqing, China; 2 College of Animal Science and Technology, Southwest University, Chongqing, China; 3 College of Computer and Information Science, Chongqing Normal University, Chongqing, China; 4 College of Life Science, Chongqing Normal University, Chongqing, China

## Abstract

*Pasteurella multocida* can infect a wide range of host, including humans and animals of economic importance. Genomics studies on the pathogen have produced a large amount of omics data, which are deposited in GenBank but lacks a dedicated and comprehensive resource for further analysis and integration so that need to be brought together centrally in a coherent and systematic manner. Here we have collected the genomic data for 176 *P. multocida* strains that are categorized into 11 host groups and 9 serotype groups, and developed the open-access *P. multocida* Database (PamulDB) to make this resource readily available. The PamulDB implements and integrates Chado for genome data management, Drupal for web content management, and bioinformatics tools like NCBI BLAST, HMMER, PSORTb and OrthoMCL for data analysis. All the *P. multocida* genomes have been further annotated for search and analysis of homologous sequence, phylogeny, gene ontology, transposon, protein subcellular localization and secreted protein. Transcriptomic data of *P. multocida* are also selectively adopted for gene expression analysis. The PamulDB has been developing and improving to better aid researchers with identifying and classifying of pathogens, dissecting mechanisms of the pathogen infection and host response.

## Introduction

*Pasteurella multocida* are a group of gram-negative coccobacillus-shaped organisms that are well known for causing diseases in a wide range of birds and mammals, including humans and economically important animal species (1). Infections by *P. multocida* can cause serious diseases, such as fowl cholera, swine atrophic rhinitis, bovine haemorrhagic septicaemia and lower respiratory tract infections (pneumonia and pleuritis), rabbit snuffles, human wound abscesses and meningitis following cat- or dog-inflicted injuries (2). *P. multocida* infections also lead to big economic losses to animal husbandry in worldwide. There are 5 serovars, A, B, D, E and F, which have been classified based on different capsular antigens (3, 4), and 16 somatic types based on the lipopolysaccharide antigens variation (5).

The first complete genome sequence of *P. multocida* was sequenced from strain Pm70, which was isolated from avian host (6). So far, 176 *P. multocida* genomes have been released in GenBank (https://www.ncbi.nlm.nih.gov/genome/genomes/912), including 57 complete assemblies. *P. multocida* genomes are characterized to be between 2.05 and 2.70 Mb in size and comprise a single circular chromosome with a G + C content of between 36.9% and 44.0%. The genomes have been used to identify important similarities and variations between strains, as well as phylogenetic relationships (2). Furthermore, genomic data also provide important references for further studies on mechanisms of pathogenesis, host specificity and virulence (7–9).

The increasing genome data of *P. multocida* provide a valuable resource for scientific research. However, to date no comprehensive and specific platform that serves genomic data and analysis for *P. multocida* has been developed. The *P. multocida* PubMLST database is a platform comprising a sequence/profile definitions database, which contains allele sequence and multilocus sequence typing profile, and a isolates database, which supplies provenance and epidemiological information (10). Here, we report the PamulDB, a resource for *P. multocida* genomics and comparative genomics and providing researchers not only functional annotation of genes and gene products, but also extensive information for gene family, phylogeny, gene ontology, gene expression, protein domains and subcellular localizations, as well tools for browsing, searching and analyzing data.

## Datasets and methods

### System implementation

The PamulDB is developed on a platform mainly powered by Linux CentOS Server 7, PostgreSQL 9.6, Apache 2.4, PHP 7.1 and BioPerl 1.6, which are running on a computer cluster composed of Dell PowerEdge R920. The implementation of the database integrates and harnesses three well-interconnected softwares, Chado (11), JBrowse (12) and Drupal (http://www.drupal.org), which make up the main architecture of the database. The Chado schema managed by the PostgreSQL stores all genomic data and forms the core of the whole system. On this basis, we developed program methods to control data communications between the Chado and applications, including the JBrowse (12) and Drupal.

The Chado is a relational database schema and widely used to manage biological data from a wide variety of organisms. Chado makes extensive use of controlled vocabularies including sequence features to type all entities in the database. The controlled vocabulary is easily extensible to new data types. All genomic features, such as chromosome, scaffold, gene, mRNA and polypeptide, are stored in a feature table, with the type provided by Sequence Ontology (SO, http://www.sequenceontology.org). Formatted genomic features are loaded into the database in batch using a built-in Perl script of Chado, the gmod_bulk_load_gff3.pl.

The JBrowse is a fast and scalable genome browser built completely with JavaScript and HTML5. As one of the core components of the GMOD project (http://gmod.org/wiki/Main_Page), JBrowse is widely used and combined to databases for displaying genome annotations. To browse *P. multocida* genomes, gene models and annotations are retrieved from the Chado schema using the Bio::Chado::Schema Perl API, and then transferred into JSON format so that can be explored with the JBrowse.

The Drupal is a popular content management and open source software system, and has been widely used to build web-based portal for biological databases (13–15). To display and operate data in Chado with Drupal, an Application Programming Interface (API) was developed with PHP. The API is composed of a set of functions, which are capable of receiving and parsing commands sent from the Drupal, and then executing commands to communicate with Chado, finally returning data to Drupal, where the data will be further processed and displayed to users. Bootstrap framework (http://getbootstrap.com) is integrated with Drupal to friendly display data supporting both desktop and mobile devices. Besides, tables in the database are rendered with DataTables (https://datatables.net), which is a plug-in for the jQuery JavaScript library and a highly flexible tool that adds advanced features to any HTML table.

### Data and processing

176 genomes of *P. multocida* strains have been released in GenBank by 20 November 2018, including 4 genomes from strains of CQ2, CQ6, B and F, which were sequenced and submitted by our laboratory. The 176 *P. multocida* strains are composed of 3 gallicida subspecies, 15 septica subspecies, 46 multocida subspecies and 112 strains without subspecies assignment. Meanwhile, 47 strains have been serotyped into eight serovars, A, A1, A3, A5, B, B2, D and F, respectively. At present, the PamulDB has recorded 12 429 genomic assemblies, 364 095 genes, 356 666 proteins and 7430 non-coding RNAs (tRNA and rRNA) from the 176 strains (Table 1).

**Table 1:** Summary of *P. multocida* strains and major data types available in PamulDB

	No. of records														
	Subspecies					Serotype									Total
	*gallicida*	*septica*	*multocida*	unclassified		A	A1	A3	A5	B	B2	D	F	NA^a^	
Strains	3	15	46	112		13	5	5	1	2	17	1	3	129	176
Assembly	502	301	7166	4460		244	84	64	6	39	732	1	7	11 252	12 429
Gene	4274	32 292	91 544	235 985		27 604	10 532	10 722	2 176	4 023	36 903	2 256	6 259	263 620	364 095
Protein	4165	31 495	89 007	231 999		26 821	10 223	10 409	2102	3962	35 942	2183	6111	258 913	356 666
ncRNA^b^	109	797	2 537	3 987		784	309	313	74	61	961	73	148	4707	7430
Transposon	101	570	1740	4129		516	192	181	35	72	646	38	123	4737	6540
Gene ontology															
Molecular function	708	740	760	760		734	716	722	712	679	719	43	709	771	771
Biological process	441	471	504	500		472	446	448	443	427	447	38	440	513	513
Cellular component	44	45	51	53		45	44	47	45	43	45	9	44	55	55

^a^Not available; ^b^tRNA and rRNA.

*P. multocida* genomes in GenBank format are downloaded and transferred into the Generic Feature Format version 3 (GFF3; https://github.com/The-Sequence-Ontology/Specifications/blob/master/gff3.md) according to SO (16) using bp_genbank2gff3.pl (https://metacpan.org/pod/distribution/BioPerl/bin/bp_genbank2gff3). For genomes that have not been annotated, protein-coding genes are predicted using Prodigal (17). Ribosomal RNA genes are identified with INFERNAL (18), RNAmmer (19) and homologous search with BLASTN (20). Transfer RNA genes are predicted using tRNAscan-SE (21). Subsequently, gene models identified with different methods are integrated with GLENA (http://sourceforge.net/projects/glean-gene) and finally transferred into GFF3 format so that can be easily loaded into Chado schema.

Newly predicted proteins are functionally annotated using BLASTP (20) searching against the Swiss-Prot and TrEMBL databases in UniProt (22). All proteins are also searched against SMART (23) and PFAM (24) databases to identify protein domains. Meanwhile, proteins are searched against InterPro (25) database using InterProScan (26) to identify Gene Ontology (GO). Totally, 188 343 proteins are annotated with 1339 unique GO terms, including 771 molecular function terms, 513 biological process terms and 55 cellular component terms (Table 1).

Protein families are identified from all proteins of *P. multocida* with OrthoMCL v2.0.9 (27). The 356 666 protein sequences are firstly all-to-all aligned using BLASTP (20), results of which are then parsed and filtered with maximum E-value of 1E-6 and minimum overlap of 50%. Subsequently orthologous and paralogous relationship are built from the BLAST results using MCL with default parameters. Finally, 354 502 proteins are grouped into 3731 families and left 2164 proteins as strain-specific singletons.

Genomic phylogeny is built with SNP sites detected in all single-copied orthologs from genomes that are in scaffold and chromosome status. All protein-coding genes from the complete genomes are all-to-all aligned using BLASTN (20) with E-value of 1E-6. Orthologous and paralogous groups are built from BLASTN results using the OrghoMCL and screened for single-copied orthologs with overlap cutoff of 60%. Totally, 310 orthologous groups from 128 strains are selected and multiply aligned respectively using Clustal Omega (28) and then conjugated, result of which are then used to detected SNPs with a Perl script. Subsequently, 16 813 SNP sites are used to build phylogenetic tree using FastTree (29) with default parameters.

Transposable elements (TEs) are predicted with RepeatMasker version open-4.0.7 (http://www.repeatmasker.org). Genome sequences are searched against Repbase Update 20.11 (30) by running RepeatMasker and RepeatProteinMask with default parameters, respectively. Totally 6540 TEs have been identified, including 1242 DNA transposons, 2016 LINE elements, 746 LTR retrotransposons, 2496 SINE elements and 30 repeat regions (Table 1).

Transcriptome data of *P. multocida* are downloaded from GenBank GEO DataSets. There are two Serires, GSE10051 and GSE12779, that can be matched to released genome. Gene expression data are retrieved from the datasets in XML files and matched to genome to make Wiggle Track Format (WIG; http://genome.ucsc.edu/goldenPath/help/wiggle.html), which are then transferred into bigWIG (http://genome.ucsc.edu/goldenPath/help/bigWig.html) using program of wigToBigWig (http://hgdownload.soe.ucsc.edu/admin/exe/linux.x86_64/wigToBigWig) and integrated into the JBrowse.

Besides, protein localizations are predicted using PSORTb 3.0 (31), which was developed to identify subcellular localizations for prokaryotic proteins by assigning a probable localization site to a protein given an amino acid sequence alone. For gram-negative bacteria, probable values are calculated for localizations of cytoplasm, cytoplasmic membrane, extracellular part, outer membrane and periplasmic region, respectively. In a result, 286 031 proteins have been automatically assigned to a subcellular localization, including 1812 extracellular proteins.

## Results

### Search and browse data

**Figure 1:**
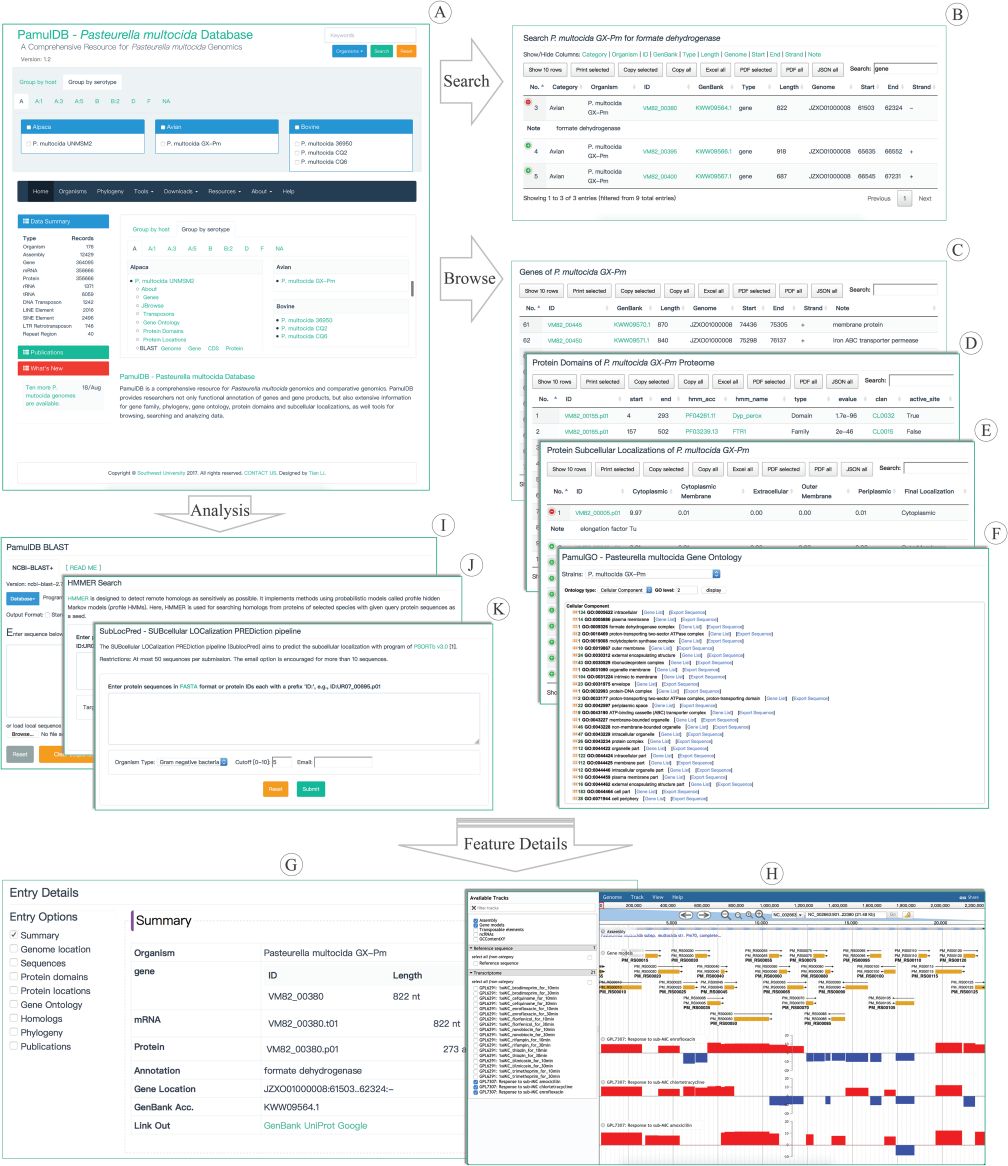
Highlights for the homepage, tools, overview pages and entry details of PamulDB. (**A**) The homepage with quick accesses to search, analysis tools and data for each strain. (**B–E**) An example of searching results and browsing genes, protein domains and subcellular localizations. (**F**) PamulDB gene ontology browser shows GO terms at multiple levels and can export sequences for each level. (**G**) Details for a genome feature, including data summary, genome location, sequences, protein domains, protein locations, gene ontology, homologs, phylogeny and publications. (**H**) PamulDB genome browser is built with JBrowse and used to graphically view genome features. (**I and J**) Analysis tools of BLAST and HMMER used for searching homologous sequences. (**K**) Analysis tool of SubLocPred used to predict protein subcellular localizations.

*P. multocida* strains are categorized by serotype and host so that can be quickly accessed from the front page (Figure 1A) and analysis tools that require strain selection. Currently, 11 host groups and 9 serotype groups have been categorized for the 176 strains, respectively. 47 out of 176 strains have been serotyped into 8 serovars including A, A:1, A:3, A:5, B, B:2, D and F. All strains have been assigned host information except for PMTB2.1.

A powerful search tool is designed on top right of every web page (Figure 1A). The tool allows users to perform queries on single and multiple strains, and supports searching for keywords in multiple types, including sequence identifier, description and annotation. Besides, the search tool can also be used to fetch sequences with given location information in format of `ID:start..end:strand’, like LGRE01000001:1248..1988:-. Search results are presented in a table that can be sorted and filtered for further searches, and can also be copied, printed and exported in formats of XLS, PDF and JSON (Figure 1B), respectively.

Genomic features, like genes, TEs, protein domains, protein subcellular localizations, can be directly accessed from links under each Species name on the front page. Clicking on the link of genes, TEs, protein domains and protein subcellular localizations brings you to a responsive table, which can be searched, filtered, selected, copied and exported to JSON, XLS and PDF, respectively (Figure 1C–E). Besides, the GO browser (PamulGO) allows users to list genes and export sequences based on GO terms (Figure 1F). Clicking on a feature ID in each feature table and the PamulGO brings you to a responsive webpage, where details of each feature, like sequence length, molecular weight, genomic position, GO, publication, gene family, phylogeny, protein domain and subcellular localization, can be selectively viewed (Figure 1G). Genome features can also be viewed with the JBrowse (Figure 1H) that supports visualizing feature position, sequence, G + C content, functional annotation and relationship among features. Besides, transcriptomic data of strain Pm70 are also available in the JBrowse at address of https://biodb.swu.edu.cn/bio/jbrowse/?data=db%2Fpamuldb%2Fpamul_multocida_Pm70.

The whole genome-based phylogeny of *P. multocida* strains can be viewed via the `Phylogeny’ link on the main menu of the website. PhyD3 (32) is utilized to display the phylogenetic tree in an interactive way so that the tree can be easily changed and export to a figure.

### Analyze data

PamulDB provides two tools for analyzing homologous sequences. One is BLAST (Figure 1I) which is built with NCBI BLAST+ 2.7.1 (20) and supports search against multiple databases. The query can be both sequences in FASTA format and identifiers of sequence in the database. BLAST results are given in standard and tabular formats from which outputs can be further searched, ordered, filtered, printed and exported in multiple formats. Besides, both matched targets and regions can also be directly exported. The other homologous search tool is HMMER (Figure 1J) that implements method using probabilistic models called profile hidden Markov models (33). Similarly, the results of HMMER are also listed in a versatile table, with which data can be further sorted, filtered and exported. Furthermore, PamulDB also provides tool to predict subcellular localization for proteins. The SubLocPred (Figure 1K) is built with PSORTb 3.0 (31) that supports three models, gram-positive bacteria, gram-negative bacteria and archaea.

### Download data

Genomic sequences, including assembly, gene, CDS, protein, rRNA and tRNA, can be directly downloaded in bulk via the `Downloads’ drop down menu in every webpage (Figure 1A). Clicking the `Datasets’ under the `Downloads’ menu brings you to a datasets webpage, where *P. multocida* strains are grouped by host and serotype, respectively. In each group there is a table providing not only download hyperlinks but also basic information for the dataset of each strain.

## Conclusion and future perspective

*P. multocida* are a group of economy- and health-important pathogens that are prevalent worldwide and actively studied. More and more *P. multocida* genomes have been submitted to GenBank, which is a general and comprehensive resource but has not provided specific and comprehensive functions for analysis on *P. multocida* data. Actually, there is no database that specially serves genomic data search and analysis for *P. multocida* yet. Here, the PamulDB provides an efficient and effective way to manage and analyze genomic data of *P. multocida*, as well as friendly interfaces and tools to perform search and analysis. With these functions, PamulDB can be used to help researchers with classifying *P. multocida* strains, dissecting mechanisms of pathogen infection and host response, as well as developing quarantine methods and tools.

In the future, we will continue to incorporate more genomes and additional data from the pathogen, plasmid and host into the PamulDB. Studies on *P. multocida* infections, host responses and epidemiology have produced massive data (34–38). Integration of these data into PamulDB will provide more references for further researches. Besides, we are developing more tools for data analysis and mining, such as genome structure variations, pathogen diagnosis and classification with given specific sequences, gene localization and map drawing, and so on. Furthermore, PamulDB have provided basic external links to other public databases, including GenBank, UniProt, SMART and PFAM. We are developing functions to retrieve further information from more public resources, like data of protein interactions and structures, which will be more help with understanding the pathogenesis of *P. multocida.*

## Notes

**Database URL:** https://biodb.swu.edu.cn/pamuldb
